# [Retracted] Downregulation of uPA/uPAR inhibits intermittent hypoxia-induced epithelial-mesenchymal transition (EMT) in DAOY and D283 medulloblastoma cells

**DOI:** 10.3892/ijo.2026.5902

**Published:** 2026-06-08

**Authors:** Reshu Gupta, Chandramu Chetty, Praveen Bhoopathi, Sajani Lakka, Sanjeeva Mohanam, Jasti S. Rao, Dzung H. Dinh

Int J Oncol 38: 733-744, 2011; DOI: 10.3892/ijo.2010.883

Following the publication of the above article, a concerned reader drew to the Editor's attention that there were anomalies associated with the cellular images shown in Fig. 1A; essentially, groupings of cells/cellular features appeared to be strikingly similar in appearance, looking internally within four of the eight data panels in this figure. A similar phenomenon of strikingly similar features was observed in the majority of the panels showing spheroid formation assay data in Fig. 8A. Moreover, regarding the western blots shown in Fig. 2B for the D283 cell line, the protein bands shown for E-cadherin for the normoxia and hypoxia cells [the (N) lanes] were strikingly similar, suggesting that the gels in this figure may have featured inappropriate editing. Finally, some of the western blot data featured in Fig. 5C were very similar to data that had appeared in a paper by the same authors published in the journal *PLoS One*.

After having conducted an internal investigation of the data in this paper, the Editor of *International Journal of Oncology* has decided that this article should be retracted from the journal on the grounds of a lack of confidence in the data. The authors were asked for an explanation to account for these concerns, but the Editorial Office did not receive a reply. The Editor sincerely apologizes to the readership for any inconvenience caused, and we thank the reader for bringing this matter to our attention.

